# The ambiguity of working class interests in Brazilian road transport: the case of self-employed truckers

**DOI:** 10.3389/fsoc.2025.1623186

**Published:** 2025-08-14

**Authors:** Jörg Nowak

**Affiliations:** Institute of International Relations, University of Brasilia, Brasilia, Brazil

**Keywords:** truckers, Brazil, self-employment, working class interests, transport

## Abstract

In many theories on working class power, the formation and existence of the interests of workers is assumed as a given, although it requires political processes for these interests to emerge at all. While this process often remains hidden behind assumptions that trade unions simply represent given economic concerns of workers, the ambiguity and fragility of the formation of working class interests is visible in situations in which the status of the wage earner and/or the self-employed worker is not clear cut or ambiguous, and these situations tend to appear more often in countries of the Global South. Occupations in transport like taxi drivers and truckers are traditionally prone to exhibit these ambiguous situations. This article explores one of those situations with the example of the fragility of the formation of working class interests among Brazilian truckers. The majority of this occupational group consists of self-employed truckers, and in spite of their shared interests with the smaller group of formally employed truckers, they tend to mobilize and organize together with employers, i.e., transport companies. I will explain why this is the case with reference to historical examples and the specific concerns of self-employed truckers and transport companies.

## 1 Introduction

In many theories on working class power, the formation and existence of the interests of workers is assumed as a given, although it requires political processes for these interests to emerge at all. While this process often remains hidden behind assumptions that trade unions simply represent given economic concerns of workers, the ambiguity and fragility of the formation of working class interests is visible in situations in which the status of the wage earner and/or the self-employed worker is not clear cut or ambiguous, and these situations tend to appear more often in countries in the Global South. Occupations in transport like taxi drivers and truckers are traditionally prone to exhibit these ambiguous situations. This article explores one of those situations with the example of the fragility of the formation of working class interests among Brazilian truckers. The majority of this occupational group consists of self-employed truckers, and in spite of their shared interests with the smaller group of formally employed truckers, they tend to mobilize and organize together with employers, i.e., transport companies. I will explain why this is the case with reference to historical examples and the specific concerns of self-employed truckers and transport companies.

Numbers regarding registered employed and self-employed truck drivers show a number of 546,499 self-employed drivers and of 344,231 formally employed drivers in Brazil (ANTT 2023). The main tendency in terms of worker representation in the road transport sector in Brazil consists in a separate organization of self-employed and formally employed truck drivers, often together with other occupational groups. This general tendency is reinforced by the fact that parts of the employers and parts of the trade unions of self-employed drivers are organized in the corporatist body CNT (Confederação Nacional de Transporte) which also represents actors of other transport modes. The joint organization of employers and self-employed truck drivers already sets the tone: While since the 1980s self-employed truck drivers in Brazil increasingly organized in separate trade unions without other occupational groups like taxi drivers with whom they used to share the same trade union, their mobilizations like national strikes have often been organized with the support of employers, and/or under the leadership of organizations who represent both employers and self-employed truckers. Self-employed truck drivers are usually the most militant group in those strikes, but representatives of employers often dominate the list of demands and the negotiations with government which accompany or follow strikes. Thus, there is strong collusion between employer interests and the interests of self-employed truck drivers. This collusion is not just a capture of genuine workers’ interests by employers, although this is certainly one aspect, but it is also based on common grievances of both groups which are not shared to that extent by formally employed drivers: Both transport companies and self-employed truckers are affected by low freight prices and high diesel prices which have been the main grievances of protests in the sector at least since the mid-1970s.

In the first part of this text, I will introduce the methodology used. Then, I will address how class theory approaches the question of self-employment and ambiguous class positions. This is followed by a section that explores the specific configuration of class and labor in the Brazilian road freight sector. This is followed by a section that presents the historical tendencies of organization of actors in large mobilizations of truckers. In the next part, I will discuss the findings in light of the theories introduced earlier, which is followed by a conclusion.

## 2 Methodology

Apart from the study of documents, statistics and existing academic literature, the research for this article is based on 15 interviews that have been conducted between February 2021 and February 2023 with executives at freight platform companies, trade union officials of truck driver unions, truck drivers and experts at state agencies concerned with transport. Of those interviews, 10 were led with truck drivers and union officials of truck drivers unions, three of the interviewed persons were from management of road cargo platforms, one interview was with a researcher specialized on the Brazilian road cargo sector, and one interview was conducted with two officials from the Brazilian state agency of Terrestrial Transport *Agência Nacional de Transportes Terrestres* (ANTT). The interviews with corporate representatives, the expert researcher and the officials of ANTT were led via internet or phone. Of the 10 interviews with truck drivers and union officials eight were led face to face, one via telephone and another one via internet. Interviews had a length varying between 15 and 80 min, with a medium length of about 45 min.

In order to gather data on protests of truckers, I made archival research in the online archive of the Brazilian business newspaper *Valor Econômico*. Data on the development of the Brazilian road transport sector was gathered and evaluated based on the online database RNTRC Panel Data of ANTT (2023).

## 3 The theory of the empirical, or the empirics of theory: class and class identity of formally self-employed workers

Self-employment, petty commodity production, and disguised waged work are often hard to distinguish. This section touches both on empirical and theoretical questions since both are very much related, and it is the lack of transparent information on empirical reality and the lack of clear-cut distinctions between groups and actors which makes theorization challenging and quite complex.

Self-employment and petty commodity production figure in the work of Marx as belonging primarily to the intermediate classes which are often leftovers from pre-capitalist forms of production. Regarding the transition of handicraft production to manufacture, Marx underlines in the *Grundrisse* that peasants who did spinning and weaving as a secondary activity were hired by mercantile capital to produce for them full time, eventually becoming wage laborers or retaining their own means of production: “He (the Merchant, J.N.) buys their labor and takes their property first in the form of the product, and soon after that of the instrument as well, or he leaves it to them as *sham property* in order to reduce his own production costs.” (1973, 510; emphasis in the original). This transitional form between independent rural production and manufacture reappears with some frequency in a number of different branches. In this sense, it is questionable if workers who sell goods or services based on small-scale property of means of production, are really independent producers or rather workers who own sham property as Marx put it.

In the context of small production units in India, Harriss-White (2014) claims that the bulk of informal work in India, representing about 90 per cent of employment in India, is petty commodity production (PCP) with some amount of independence from buyers, i.e., Harriss-White claims that most of this PCP is not disguised wage work. PCP is characterized by the fact that there is no capital accumulation, and is therefore distinguished from capitalist small and medium enterprises. The claim of Harriss-White has been criticized fiercely by Bhattacharya (2014) who claims that Harriss-White tends to do away with the category of labor.

In a historical account around the introduction of labor legislation in the Indian garment sector in the 1940s and 1950s Dietrich Wielenga (2020) describes how the restriction of labor law to some groups was established by court proceedings, and that therefore the state has been central in establishing who are informal and who are formal workers. Furthermore, she also underlines the permanent changes in legal stipulations around garment work, i.e., employers dismissed workers and set them up as independent suppliers in order to circumvent labor law in a large number of cases. This would be a classical case of disguised wage labor in order to circumvent certain legal obligations of employers, representing the sham property that Marx mentions in the *Grundrisse.* The independence of those garment workers would therefore be a mere juridical appearance, since they are formally self-employed, but de facto dependent on the buyers of their products who used to be their employers a short while ago.

The two examples from India show that there is sufficient controversy about how to establish neat distinctions between the different categories of waged workers, disguised waged workers and petty producers. This is more so the case in countries where informality is widespread, and numbers about certain employment relations and economic activities might not be readily available across all relevant areas.

We can now apply this theoretical debate to the sector of road transport. Marx argues in the *Grundrisse* (Marx, 1973, p. 522f) and in *Capital Vol. 2* that the transport of a product to its point of sale is part of the process of production, and an activity that produces surplus value. Therefore, “The costs of production would resolve into the labor time objectified in the direct production process + the labor time contained in transport” (Marx, 1973, p. 522). Following Marx, workers in commodity transport are part of the industrial working class who produce surplus value and participate in the production of the good to be sold. Across the globe, while some percentage of workers in road transport are formally employed, a significant amount works as owner-operators which does not seem to be a transitional situation since it persists since the beginning of motorized road transport of goods. Therefore, drivers in road transport do not represent that part of the intermediate classes who do unproductive labor but they are part of productive workers. In this respect, they can be compared to small-propertied producers (Hodges, 1961, p. 29). In the Theories of Surplus Value, Marx emphasizes that peasants and artisans who own means of production are neither productive nor unproductive (1952, 191) since they fall outside the capitalist mode of production. But also in this case, Marx mentions the farmer “who as a result of a mortgage, has ceased to be the real owner of his land and remains only its nominal owner” (Hodges, 1961, p. 33; Marx, 1952, p. 193f), this farmer being a worker in the end.

This situation of sham property, or being only a nominal owner, applies to truck drivers since they usually buy the truck from a vendor and then pay installments for the vehicle over a number of years, similar to the mortgage paying farmer. The truck driver usually pays the installments for the vehicle to another capitalist or to a bank from whom he received a credit in advance. But Hodges underlines the following: “The relation of employer to worker, for example, is a basic or direct form of exploitation, whereas that of landlord to tenant-farmer and that of bankers to small-propertied producers, is a derivative or indirect one” (Hodges, 1961, p. 34). Thus, even if small-scale owners of means of production are highly dependent on capital, this situation remains with a semblance of independence, since small-scale owners of means of production do not have to follow orders in a day-to-day fashion, even if in the end their margin of independent decision-making is rather small.

This appearance of independence has then consequences for class-formation. The question of identification of workers as being part of the working class came up in various debates, among other regarding the class identity of white collar employees. Kautsky (1971) raised this issue in the following words: “The time is near when the bulk of these proletarians will be distinguished from the others only by their pretensions. Most of them imagine that they are something better than proletarians. They fancy they belong to the bourgeoisie, just as the lackey identifies himself with the class of his master” (Kautsky, 1971, p. 40). As has been highlighted by Poulantzas (1975) and Przeworski (1985), the interpretation that workers make of their social and political position, is part of the process of class formation. “Classes as historical actors are not given uniquely by any objective positions” (Przeworski, 1985, p. 66). If classes are effects of struggles, as Przeworski assumes, then the outcomes of those struggles are not determined exclusively by the positions of persons in relations of production, but politics and ideology come in. In this sense, against an economistic understanding of class, “ideological and political relations” assume “the status of objective conditions of class struggles” (Przeworski, 1985, p. 68). Therefore, as E. P. Thompson points out, class experience is determined by the relations of production, but class consciousness is not (Thompson, 1963, p. 9f).

In this sense, the relations of production form a structure of choices that actors can make: “Social relations are objective with regard to the processes of class formation only in the sense that they structure the struggles that have the formation of classes as their potential effect” (Przeworski, 1985, p. 73). In the case of occupational groups, this means that their class identity will be influenced by their economic situation, but also by political and ideological elements. In this sense, class struggles take place between classes but are also “struggles *about* class formation as well as struggles *among* organized class forces” (79), in other words conflicts about the definition of groups as being workers or self-employed or entrepreneurs, and also conflicts between trade unions and associations that represent professional groups.

The approach to see class formation as a problematic and contested process is at odds with theories about working class power resources that assume the interest position of workers are evident (Schmalz et al., 2018). If for example logistical power of truck drivers is useful to block roads and the overall economy, it is less clear why truck drivers rarely succeed to attain their demands (Nowak, 2021). I claim that answers to this mismatch can be found if we look at processes of class and interest formation.

As is known from conflicts between trade unions, conflicts within professional or occupational groups about representation are rather common. To put it slightly differently, “classes-in-struggle are an effect of struggles about class” (79), and it is not guaranteed that occupational groups will organize as classes, or rather in cross-class coalitions. The phenomenon of many political parties representing cross-class alliances is regularly also found in the sphere of economic representation.

Among self-employed truck drivers, ideologies about independence and economic self-sufficiency are a frequent element, despite the dependence on a large number of external actors (Fleming, 2000, pp. 176–178): primarily, truck drivers are dependent on transport companies that hire them and/or shippers that need transport, since demand for transport services is closely related to the demand for the goods that are being transported. Second, truckers depend on fluctuations in freight prices. Third, truckers depend on the volatility of prices of inputs, primarily fuel, but also tires, car repair, vehicle parts and others. Fourth, demand is often seasonal, due to harvest seasons, a crucial element in the case of Brazil.

The question that I pursue here and that has not been covered by any research so far, is how this slightly ambiguous class position of road transport workers plays out in the forms of organization of self-employed truck drivers in Brazil who represent two thirds of all truck drivers in that country. In this sense, I assume that class formation is a process with open outcomes which is on the hand influenced by the material situation of truck drivers, and on the other hand by political and ideological aspects. My claim here is that the specific market situation of self-employed truck drivers provides strong incentives for them to identify with employer interests instead of the interests of formally employed truck drivers. My claim here is, in tension with the emphasis of Przeworski on ideological and political aspects of class formation, that it can be material short- to mid-term common interests that can lead transport workers to enter into alliances with employers. This does not claim that political and ideological aspects of class formation are irrelevant, but underlines the entanglement of material interests with those political and ideological moments of class formation.

## 4 Class and labor in the Brazilian road transport sector

In order to illustrate the question with our example at hand, the legal definition of a self-employed trucker in Brazil is that he or she owns between one and three trucks. Even if she owns only one truck, she might rent it out to other drivers, providing access to the means of production for a fee. Furthermore, the difference between a self-employed trucker and a small company might be further diluted since many transport companies who hire self-employed truckers urge them to get registered as a small company because this would reduce the risk of those companies to be held responsible for disguised wage employment (Silva and França, 2004; Huertas, 2013, p. 157).

Many transport companies in Brazil employ a certain number of formally employed drivers, and hire self-employed drivers in addition to fulfill other transport orders, for example in peak times.^1^ The self-employed truckers are then in fact often dependent on the transport companies, and are in fact disguised waged workers, not very different from Uber drivers or food delivery workers. In the statistics, they might then even appear as small companies in those cases where they follow certain instructions by transport companies.^2^ This situation of disguised waged work and dependency of self-employed truckers on transport companies is reinforced by the fact that self-employed truckers do not have the legal right to close transport contracts with shippers, and therefore they require an intermediary like a transport company in order to get orders.^3^ These intermediaries traditionally take high fees of about 20–40% of the freight price from self-employed truckers.

We therefore are able to identify the problems around the categorization of workers and the identification of de facto dependent employment: There is a lack of data and research on the road freight transport sector in Brazil, and the fact that many small companies work as agencies or intermediaries who have no interest to disclose the fact that they facilitate disguised waged work does not create optimal conditions to assemble this information in a systematic manner. The high number of self-employed truckers creates difficulties in investigating the nature of their business, i.e., if they rather rent out trucks to others or are rather de facto dependent on a disguised employer.^4^ Usual criteria of de facto employment include the amount of orders which are coming from the same shipper or transport company. The most comprehensive data comes from the CNT which does not disaggregate the numbers (CNT, 2023)—since employers dominate the agenda within CNT, there is also not much interest to provide more detailed data which would fill the blanks. University departments who dig deeper into transport issues are also notorically close to the agenda of employers and have not provided much data in this respect. For example, we do not know exactly how many of the registered self-employed truck drivers are economically active, and if so, to what extent.^5^ We also do not know how many of the registered transport companies are de facto self-employed truck drivers. Given that out of 295,283 registered transport companies in 2022 24.6% are micro-entrepreneurs, we can assume that there are about 75,000 self-employed truck drivers among those transport companies (ANTT, 2023).

It in this respect that the empirical issues on the ground and the theoretical questions how to categorize economic activities are closely intertwined and hard to disentangle. We have seen that the larger group of truckers in Brazil, self-employed truckers, might be self-employed, disguised wage workers, or small entrepreneurs who do not accumulate capital. The usual assumption in the literature and media is that the largest part of self-employed truckers are disguised wage workers but we have no solid data to prove this. Protest actions in forms of protests and strikes are dominated by the tendency of common action of transport companies with self-employed truckers (Huertas, 2021; Nowak, 2021). While this is counter intuitive toward the claim of most of self-employed truckers being de facto disguised waged workers, I will demonstrate that there are material issues that create a larger commonality of their grievances with transport companies than with formally employed truckers.

In order to understand this process of interest formation, I will employ the distinction between interests, preferences and strategies established by Arnholtz and Refslund (2024). They start with raising the ambiguity of the nature of workers interests since these are not homogeneous, and might differ between certain social characteristics as “gender, ethnicity, age and citizenship” or skill differentiation (Arnholtz and Refslund, 2024, p. 16). We can add to this the difference between short-term and long-term interests, i.e., between receiving a higher income and consequences for the health of workers. Interests then become manifest as soon as they are expressed as preferences which includes a social and political process of selection among given interests. “Establishing, maintaining, and developing a collective that can mobilize and utilize resources inevitably involves the formation and re-formation of preferences understood as the interests that are prioritized and articulated.” (Arnholtz and Refslund, 2024, p. 17) The formation of strategies then represents a further step of selection during which workers calculate which preferences realistically could possibly be achieved: “Distinguishing between preferences and strategy allows us to recognize actors’ reflexivity about their own and other actors’ capacities” (Arnholtz and Refslund, 2024, p. 18).

It is this process of a creation of interests, preferences and strategies which represents the different steps of the process of class formation that Przeworski theorizes. Initial interests are filtered according to pressing needs of actors, according to their opportunities for action, and their beliefs and ideas. The raw material of objective interests is therefore formed by the actors themselves and the social and political environment in which they act. This disaggregation of the notion of interests is useful to understand that the formation of working class interests is a complex process which will be influenced by social and political processes and by the context that renders some strategies more likely to be successful and might be more prejudicial for other strategies, thereby having a decisive influence on the formation of those interests. In this way, interests can be understood not as something given, but rather as the product of a process of selection: Interests therefore appear to us mostly in the form of strategies of actors, thus after the process of selection between interests and preferences has already taken place. What we can identify are the strategies and the multiplicity of interests might be hidden behind the more overt strategies.

## 5 A short history of the organization of interests in Brazilian road freight transport

The history of the organization of actors in the road transport sector shows a threefold type of organization: The employers organize in the organization NTC, later called NTC and Logistics, self-employed truck drivers organize jointly with taxi drivers in trade unions focused on self-employed in the transport sector, and formally employed truck drivers organize in transport unions together with other workers from the sector like bus drivers in passenger transport and administrative staff in transport. This threefold organization already underlines the tendency of a separate organization of self-employed truck drivers, while the formally employed truck drivers do not always form separate unions, and are rather less militant sections of the larger transport unions. Among self-employed truck drivers, there has been a tendency since the 1980s to split off from the joint unions together with taxi drivers and to organize in separate trade unions, although in an uneven fashion across several regions (Kapron, 2012, p. 139). During the 1980s, there emerged also several other organizational forms of self-employed truckers for whom it was often more beneficial to organize in the form of an association or cooperative in order to share tax obligations and other fees. Kapron notes that this led to some confusion and overlap between the organizational forms of trade union, association and cooperative among truck drivers. In addition, some truck drivers with more bargaining power, for example for the transport of dangerous goods, created their own separate organizations (Kapron, 2012, p. 139).

To add to the confusion, the organization Movimento União Brasil Caminhoneiro (MUBC) became an important actor since the 1990s, specifically in terms of organizing a major strike of truckers in 1999. MUBC organized self-employed truckers as well as employed truckers and was also open to the influence of transport companies. The strike in 1999 is said to have been the largest truckers’ strike so far in terms of a participation of 1.5 million truckers, and at the same time there have been multiple allegations that MUBC was serving mainly the interest of transport companies (Huertas, 2013, p. 161). The trade unions of Fenacam (Federação dos Caminhoneiros Autônomos), Fecam and Fetrabens (Federação dos Caminhoneiros Autônomos de Cargas em geral do estado de São Paulo, composed of 26 trade unions) were openly accusing MUBC and the trade union federation CGTB to which it was allied to have launched MUBC exclusively in order to receive trade union contributions which are transferred by state funds to trade unions which represent a sector and to launch political candidates via MUBC.

Some of the organizations who criticized MUBC, namely Fenacam, Fecone (Federação Interestadual dos Transportes Rodoviários Autônomos de Cargas e Bens da Região Nordeste) and Fetrabens then founded in 2012 the Confederação dos Transportadores Autônomos (CNTA), with the aim to create a national trade union of self-employed truckers (Huertas, 2013, p. 162). A special type of organization is the aforementioned CNT, a corporatist body which includes trade unions as well as employer organizations from various modes of transport. The CNT as a para-state organization across employers and workers is one of the major forces that facilitate the joint action of truck drivers and transport companies in the sector, which then often leads to a dominance of employer interests at the cost of specific interests of truckers.

Self-employed truckers’ organizations who did not join CNTA like Fecam and some others remained part of the CNT which also has in addition its own organization representing self-employed truckers, Abcam (Associação Brasileira dos Caminhoneiros), founded in 1983, which claims to represent 600,000 self-employed truckers, and became part of CNT in 2003. CNTA itself later became associated with CNT, too, in December 2017 and moved its headquarter from the South of Brazil in Curitiba to the CNT building in Brasilia (Huertas, 2021). The integration of many organizations into CNT has the effect to maintain political control of the organizations of truckers so that they remain tied to the interests of employers and at a distance to the left-leaning parts of the trade union movement, which still represents the largest part of organized workers, mainly in the Central Única de Trabalhadores (CUT). In the transport sector, the trade union Confederação Nacional dos Trabalhadores em Transportes e Logística, CNTTL, representing mostly employed truckers and workers from all other modes of cargo and passenger transport, is part of CUT since 1989 (Kapron, 2012, p. 141).

After the first large mobilization in 1999, MUBC remained at the forefront of truck drivers’ strikes for some time. Especially the early 2010s saw a series of truck drivers’ mobilizations, in parallel with a series of strikes in the construction industry and a general uptick of strikes since 2011. One can assume that this series of protests was partially motivated by a general mood of workers’ mobilization and a general boom in the economy which made workers believe there is room for negotiation. A larger strike of truckers in July 2012 saw mobilizations in 12 out of 27 federal units, and was spearheaded by MUBC as in 1999, directed against new regulations in the sector like the obligation regarding an electronic payment of truckers and longer rest times, as stipulated by the law 12.619/12 (Huertas, 2021). These protests against new regulations were mainly demands of employers and therefore CNTA accused MUBC at the time to represent employer interests. Another mobilization 1 year later, in July 2013, took place in nine federal states and in the climate of general social mobilization due to the street demonstrations that started in June 2013. MUBC was again the only organization openly supporting the strike, demanding lower diesel prices and lower road toll fees, more road security and once again changes to the aforementioned law 12,619/12. This time the federal government of Dilma Rousseff intervened against the strike, alleging suspicion of a lockout due to the participation of employers and blocked 6 million Real in the bank accounts of MUBC (Huertas, 2021).

In February 2015, another truckers’ strike took place in 12 states with 120 road blocks (Nowak, 2021), again with the same demands as in 2013, this time combined with the demand for state sanctioned minimum freight prices, a demand that was realized in 1979 already and met enormous problems in terms of implementation and control (Kapron, 2012, p. 144). During the strike in 2015, no organization claimed responsibility for the strike and the federal government of president Rousseff acted quickly to crack down against protesters, leveling fines of 100,000 Real per hour for blocking roads, but without much success (Huertas, 2021). After 7 days of strike, Rousseff approved a law that made working hours of truckers more flexible. In this way, the strike in 2015 was the first of this series in the early 2010s which led to tangible results, although questionable ones from the perspective of labor. In the 2015 strike, the government remained uncompromising regarding the demand to lower diesel prices, and only offered a 6 month freeze of the prices which was rejected by the representatives of truckers.

After Rousseff’s impeachment in 2016, the new strictly neoliberal Temer government changed the presidency of state oil company Petrobras and diesel prices started to adhere without delay to international prices. This was in stark contrast to earlier governments who only adapted the diesel prices to international prices with considerable delay. The new price policy led to a spike in diesel prices in 2018, and subsequently to an 11 day long truckers’ strike in May 2018. This time it were CNTA and Abcam who claimed responsibility for the strike which quickly developed a life on its own with more than 700 blockades and 400,000 mobilized truckers—the largest and longest truckers’ strike since 1999. The comprehensive road blocks caused enormous economic damage, and again transport companies participated in the mobilization. The federal government of Temer threatened to mobilize the military and called the strike a lockout, but finally soldiers were only used to secure the access to a small number of petrol depots. One popular demand during the strike was the reduction of taxes on diesel prices, which are to a smaller extent taxes by the federal unit and to a larger extent taxes by regional states. Negotiations dragged on, and the results of the strike were a 6 month long freeze of diesel prices, some smaller benefits, and the legally defined minimum freight prices (Nowak, 2021). Again, implementation of those prices was rather patchy, and employers subsequently were able to block the application of fines for the violation of the minimum prices in early 2020 via the Supreme Court.

Since the structural problems of the road transport market were not addressed with those measures, there were more than 10 attempts of truckers to repeat the 2018 strike, but with only moderate success. Some commentators think that the absence of transport companies incentivizing such a strike was lacking in comparison with the mega strike in 2018. Neither CNTA nor Abcam supported those new mobilizations, and alternative organizations of truckers like ANTB (Associação Nacional de Transporte no Brasil) and ABRAVA (Associação Brasileira Dos Condutores De Veículos Automotores) of which some found together in a new independent forum apart from the corporatist CNT, do not yet yield enough power to pull of a strike on its own. Nonetheless, the minimum freight prices are seen as a measure of potential benefit for self-employed truckers, and these minimum prices stand in contrast to the interests of employers in agribusiness and industry, who consequently and finally successfully tried various legal avenues to block those minimum freight prices. Employers in transport companies are split about the minimum freight prices, since some might benefit from them, and others who rather contract self-employed truckers might have to pay higher freight prices.

## 6 Discussion

In the light of the above, two questions are salient: 1. Why do self-employed truckers in Brazil tend to mobilize jointly with transport companies? 2. What are the challenges of this specific sector and what do these tell us about similar constellations of a collusion of worker and employer interests?

There are solid material motives for self-employed truckers to share more grievances with transport companies than with employed truck drivers. The main two demands which have dominated the agenda of movements of truck drivers were lower diesel prices and higher freight prices (via minimum prices established by the state). Both concern centrally the self-employed truck drivers and transport companies. Employed drivers do only benefit indirectly from those demands since they might better the situation of their employers, and therefore provide more secure jobs and offer some leeway for higher wages. Employed drivers have to rather confront their employers regarding conditions of work and wages, while transport companies and self-employed truckers appeal to the state—interestingly, there are no examples of an immediate confrontation between shippers on the one side, and transport companies and self-employed truckers on the other side. The state and the federal and state governments therefore act in substitution of the shippers which would logically be the immediate adversaries of self-employed truckers and transport companies. Thus, those two groups remain in a tactical coalition: self-employed truckers provide the mass basis for the mobilization of truckers, and transport companies help in the organization of those mobilizations, for example via the suspension of their activities in order to increase the pressure on other economic sectors who depend on transport or with financial help for the mobilizations and some limited support during strikes.

Other recurrent demands that came up during truckers’ mobilizations like road toll fees, and the access to credit for the purchase of vehicles and equipment clearly affect transport companies and self-employed truckers, and not employed truckers. Only the demands for more security on roads and better transport infrastructure in general like more paved roads and repair and catering services affect mainly drivers. A clear conflict of interest between employed drivers and self-employed truckers came up with the topic of a flexibilization of working time. In the perspective of self-employed drivers, a restriction of their working time would affect their competitive advantage in comparison with employed drivers, and in cases of a violation of working time regulation it would be the self-employed truckers who would have to pay fines, not the shippers or the transport companies that hire the self-employed truckers. While all drivers have an objective interest in shorter working hours since long working hours are responsible for most accidents which lead both to health risks and economic damage, the short- to mid-term interests of self-employed drivers might diverge from this common interest to some extent. Another conflict of interest between self-employed and employed drivers comes up in the case of taxes on diesel. A part of those taxes is used to fund social security systems which benefit employed truckers to a larger extent than self-employed ones. In other words, the greater exposure of self-employed workers toward market forces generates a different set of interests if compared to employed truckers.

Situations in which employer and workers interests overlap are more common than is often assumed. Contract farming is for example one such phenomenon which became widespread in the past 30 years (Vicol et al., 2021). In other words, processes of formalization of employment facilitate a separate interest formation of workers and employers, at least to some extent.^6^ But as a matter of fact, the formalization of employment relations is only one among many forms of how labor relations are organized on a global level. Given that at least 50% of all workers globally are not working in formalized labor relations, we can assume that processes of interest formation which create ambiguous situations in terms of the employer-worker distinction are of utmost relevance. These situations come with challenges to theories that are based on a neat distinction between employer and workers interests like the power resources approach among others (Schmalz et al., 2018; but see Arnholtz and Refslund, 2024 for a more differentiated approach). The power resources approach theorizes the power of workers that they might use against their employers. While in theory workers in cargo transport have the power to block roads, and do exercise this power in a regular fashion in Brazilian road transport, this power is usually not exercised against employers in the transport sector, but often jointly with those employers against the government and in an indirect fashion against the shippers which are producers from all economic sectors, and primarily from manufacturing, agribusiness, construction and retail.

Thus, it is not only interest formation which is not in line with the conventional idea of employer-worker relations in the Brazilian road transport sector, but also the exercise of power is not directed against employers in the same sector, but against producers in other sectors and the government. This comes on the one hand with a politicization of conflicts in the road transport sector since government is immediately involved and seen as the main addressee for demands. On the other hand, labor relations and relations of economic exploitation within the transport sector are depoliticized to some extent, and the adversaries are located outside of the transport sector. Due to the dependence of the transport sector on overall economic activity, visible in statistics that show how growth of the road transport sector follows closely overall GDP growth, identifying the main adversaries of truckers with the shippers outside of the transport sector is not completely incorrect. Thus, truckers face a triple enemy in transport companies, government and shippers which complicates interest formation. In this case, societal power can include alliances with employers from the sector where workers are located against employers in other sectors, but mediated by the state and government. The government in this respect represents capital as such against actors in the transport industry. Therefore it is no surprise that truckers see few results of strikes since even when they make alliances with employers from transport they face the power of employers from a larger number of sectors mobilized against their interests. Only a broader alliance of actors from the transport sector with other actors, like the temporary alliance with workers from the petroleum sector in the strike 2018, could lead to more sustainable results in the interest of truckers. But more long-term strategies of truckers with other parts of the union movement are usually blocked by employers in the transport sector. Plus, a considerable section of trucker unions and associations are in alliance with conservative parties and stay at a firm distance from other sections of the organized working class who are linked to left-wing political parties.

For a theory of interest formation and the power of the working class, our analysis of the sector of road freight transport in Brazil demonstrates that there might be situations in which short- and mid-term interests of self-employed workers, even if a large part of them are disguised wage workers, might rather converge with employers than with employed workers, due to their specific exposure to market dynamics. It also demonstrates some of the difficulties to reconstruct what are the “real interests” of self-employed truckers since these might differ depending on the temporal modality (short, mid or long term). For many self-employed truckers, it is not very realistic to work for long-term changes in the industry since de facto the general conditions have not seen much change since the 1970s, despite of a series of large protests, due to the constellation of power that puts actors in freight transport against capital from a large number of sectors who together wield more power than actors from road freight transport, even if these are able to temporarily block the circulation of goods. We can see here that while we might assume that objectively employed and self-employed truckers in Brazil have common interests in higher freight prices, shorter working times and a better transport infrastructure, the avenues to realize these interests take different routes or are blocked due to power complexes that block any changes. Therefore, in the process of a selection of preferences based on those interests, the preferences of employed and self-employed truckers embark on different trajectories, leading to separate and at times opposed strategies.

This constellation affects the idea of an objective antagonism between workers and employers, or workers and capital which in this case gets displaced toward an antagonism of truckers and transport companies with the “rest” of capital. But this also increases the asymmetric power relations of workers vis a vis capital since self-employed workers in transport do not confront their employers, but all sections of capital, i.e., the shippers whose freight payments are the essential financial source for the transport sector. This dependence on shippers also demonstrates that transport is not an independent sector, but relies essentially on other sectors who require transport of goods, or in the case of the financial sector, of banknotes. Some aspects of this constellation might be specific to the transport sector, but due to widespread phenomena of self-employment or contract work in sectors such as construction, the garment industry and agriculture it might have a broader relevance. Consequences on the level of political strategy are that sectoral tactics at least in the freight transport industry might have less chances than in other sectors, and it raises the question to what extent alliances between self-employed workers and employed workers are viable.

Research on joint organization of formal and informal drivers in the passenger transport sector in Uganda, for example, demonstrates that those contradictions between formal and informal workers can be overcome (Webster et al., 2021). It is probably important to analyze the specific constellations in economic sectors that determine how those contradictions play out. In passenger transport, there is usually no interest of larger shippers involved, or only in a very indirect manner, via the interest of employers that their workers are able to use transport to get to workplaces. But these specific constellations also mean that there is no unitary interest or power of workers in logistics and transport since its subsectors face radically different conditions. In Brazil, the different interests of family farmers and employed workers in agriculture, for example, have led to a split into two different trade unions, the larger Confederação Nacional dos Trabalhadores na Agricultura, CONTAG, representing 20 million workers, and the smaller Federação dos Trabalhadores na Agricultura Familiar, FETRAF-Sul which represents family farmers in Southern Brazil (Picolotto, 2014).

## 7 Conclusion

The analysis of the process of interest formation among truckers in Brazil demonstrates that employed truckers and self-employed truckers follow different paths of organization and mobilization. While self-employed truckers are the larger group with the larger potential of mobilization, they often enter into alliances with transport companies since they share more immediate demands with companies than with employed truckers. The question of market exposure is decisive here: the fact that employed truckers are less exposed to market forces, separates their demands and interests from the ones of self-employed truckers. It is this direct exposure to market prices of inputs like tires and diesel that leads to a formation of preferences which splits the strategies of employed and self-employed truckers. Therefore the formation of working class interests remains a fragile process and the smaller group of employed truckers remains isolated against the larger alliance of self-employed truckers with transport companies.

The case study also shows how these material constellations manifest in a longer political tradition of separate organizing of self-employed and employed truckers, leading to separate political identities and different strategies and alliances. Self-employed truckers have a rather sectoral identity, and their aim to be independent from transport companies and employers fuels their resistance to consider themselves as workers and part of the larger trade union movement. While these ideas and identifications take on a life of their own to some extent, they are grounded in their larger exposure to fluctuations of market prices of inputs and freight prices.

The situation in Brazilian road freight transport raises larger questions for the processes of interest formation of labor in the face of widespread informality and disguised wage labor in a large number of sectors. Future research has to investigate to what extent the situation of an ambiguity of employer and worker interests is specific to road freight transport or if it is also relevant in sectors with a similar structure of employment relations like construction or agriculture. It also raises questions about the way potential power of workers is derived in some accounts of the power resources approach from the capacity to block transport (see Schmalz et al., 2018 on logistical power, p. 115, 117), while the overall constellation of power asymmetries seems to be rather worse for labor in road freight transport than it is in other sectors. In other words, despite the higher economic power of truckers in comparison with other occupational groups due to their ability to block the circulation of goods, they might have less power to better their lot since they confront capital as such, mediated by government, rather than employers from their own sector. This reinforces earlier assumptions that a sectoral perspective regarding the power of workers provides a rather incomplete picture (see Nowak, 2021). Therefore, it might be necessary to situate labor conflicts within the larger social formation and its mechanisms of interest formation and political decisions, which requires an integral theory of workers’ power.

## Data Availability

The original contributions presented in the study are included in the article/supplementary material, further inquiries can be directed to the corresponding author.

## Footnotes

^1^Interview with trade union officer of self-employed truckers Sindicam in Santos, 25 April 2022; interview with trade union officer of self-employed truckers Sintracarpa in Belém, 14 September 2021.

^2^Interview with executive director of CNTA, 8 February 2023.

^3^Interview with officials of ANTT, 15 September 2022.

^4^Interview with officials of ANTT, 15 September 2022.

^5^Interview with officials of ANTT, 15 September 2022.

^6^Even formalized labor relations might still retain phenomena like common mobilizations of unions and employers, for example the three large mobilizations between 2017 and 2020 of European steel workers trade unions together with employers against steel imports from China.
